# Effectiveness of Resistance Training and Associated Program Characteristics in Patients at Risk for Type 2 Diabetes: a Systematic Review and Meta-analysis

**DOI:** 10.1186/s40798-021-00321-x

**Published:** 2021-05-29

**Authors:** Raza Qadir, Nicholas F. Sculthorpe, Taylor Todd, Elise C. Brown

**Affiliations:** 1 Oakland University William Beaumont School of Medicine, 586 Pioneer Dr, Rochester, MI 48309 USA; 2 Institute of Clinical Exercise and Health Science, University of the West of Scotland, Lanarkshire, UK; 3 School of Health Sciences, Oakland University, Rochester, MI 48309 USA

**Keywords:** Resistance training, Prediabetes, Metabolic syndrome, Cardiometabolic

## Abstract

**Background:**

Resistance training (RT) is an effective intervention for glycemic control and cardiometabolic health in individuals with type 2 diabetes (T2D). However, the use of RT in individuals at risk for T2D to prevent or delay the onset of T2D, and RT program characteristics that are most effective are still unknown. The purpose of this review is to determine the effects of RT on cardiometabolic risk factors in those at risk for T2D and to examine RT program characteristics associated with intervention effectiveness.

**Methods:**

PubMed, Cochrane, Web of Science, and Embase databases were systematically searched for published controlled trials that compared cardiometabolic outcomes in adults with cardiometabolic risk for those that underwent an RT intervention with those that did not. A systematic review and meta-analysis was conducted to determine the effect of RT on glycosylated hemoglobin (HbA1c), fasting plasma glucose (FPG), body fat percentage (BF%), total cholesterol (TC), high-density lipoprotein (HDL), low-density lipoprotein (LDL), and triglycerides (TG). Additional analyses examined effects of intervention duration and dietary intervention on FPG and TG.

**Results:**

Fourteen trials with 668 participants were included. For RT compared to controls, the standardized mean difference (SMD) was −1.064 for HbA1c (95% confidence interval [CI] −1.802 to −0.327; *p*=0.005), −0.99 for FPG (95% CI −1.798 to −0.183; *p*=0.016), −0.933 for TC (95% CI −1.66 to −0.206; *p*=0.012), −0.840 for BF% (95% CI −1.429 to −0.251; *p*=0.005), −0.693 for HDL (95% CI −1.230 to −0.156; *p*=0.011), −1.03 for LDL (95% CI −2.03 to −0.050; *p*=0.039), and −0.705 for TG (95% CI −1.132 to −0.279; *p*=0.001).

**Conclusions:**

RT is beneficial for improving glycemic control, BF%, and blood lipids in those at risk for diabetes. The addition of a dietary component did not result in larger reductions in FPG and TG than RT alone.

**PROSPERO Registration ID:**

CRD42019122217

**Supplementary Information:**

The online version contains supplementary material available at 10.1186/s40798-021-00321-x.

## Key Points

- Resistance training (RT) is effective for improving glycemic control and blood lipid profiles in adults at risk for type 2 diabetes (T2D) and could serve as an effective intervention to prevent or delay the onset of T2D.
- Free weight and resistance band training at intensities above 60% one-repetition maximum are effective for improving glycemic control and blood lipid profiles in adults at risk for T2D.
- A dietary component combined with RT is not more effective for glycemic control than RT alone in adults at risk for T2D.

## Introduction

Type 2 diabetes (T2D) affects over 400 million people worldwide [1], accounts for around 90% of all cases of diabetes [2], and has an estimated annual global cost of 1.3 trillion dollars [3]. This disease is linked to premature mortality and significant morbidity, primarily a result of hyperglycemia-induced cardiovascular disease (CVD) and microvascular complications including neuropathy, nephropathy, and retinopathy [4]. Prediabetes is a condition defined as elevated blood glucose (BG) levels below the level considered to be T2D and impacted over 352.1 million people worldwide in 2017 [5]. This condition often leads to metabolic syndrome, a state characterized by insulin resistance, abdominal obesity, hypertension, and dyslipidemia [6]. Metabolic syndrome and prediabetes increase the risk for developing chronic diseases such as T2D and CVD, as 5–10% of those with prediabetes progress to T2D annually [6]. Treatment strategies, such as lifestyle modifications, that address abnormal metabolic risk factors, such as glycosylated hemoglobin (HbA1c), fasting plasma glucose (FPG), and blood lipids, can reduce rates of progression to T2D [7, 8].

Intensive glycemic control has shown reductions in hyperglycemia-induced microvascular complications in T2D [9]. The gold standard for assessing glycemic control is HbA1c [10], a reliable estimate of long-term glycemic control that indicates mean plasma glucose levels over the previous 3 to 4months and is not susceptible to diurnal variations in BG levels [11, 12]. Prediabetes and T2D can be assessed through HbA1c, FPG, 2-h plasma glucose values, or 75-g oral glucose tolerance tests [13].

Blood lipid values such as total cholesterol (TC), low-density lipoprotein (LDL) cholesterol, high-density lipoprotein (HDL) cholesterol, and triglycerides (TG) are relevant to cardiovascular health in prediabetes [14]. Controlling cholesterol levels can decrease morbidity and mortality rates in individuals with prediabetes, as increased cholesterol levels are evident in individuals with prediabetes compared to individuals with normal glucose tolerance [15, 16]. Medical recommendations to utilize cholesterol-lowering medications (i.e., statins) for individuals with diabetes to control LDL levels are based on accelerated rates of atherosclerosis and subsequent coronary artery disease [17, 18]. This signifies the importance of blood lipid management in individuals with elevated BG levels.

Preventative medicine warrants the need for lifestyle modifications such as regular exercise to prevent the risk of developing these diseases [19]. Exercise can reduce insulin resistance and improve glycemic control and blood lipid profiles in those with and without T2D [20–23]. Thus, incorporating sustainable exercise modalities that improve cardiometabolic risk factors can improve health outcomes. The impact of aerobic training (AT) on health outcomes is well researched [24]. Resistance training (RT) has gained popularity for its impact on improving body composition and muscular strength and, more recently, for its role in health and disease [25]. These benefits include improved glycemic control, blood lipid profiles, and bone mineral density in healthy populations [25]. Research has supported the combined benefits of AT and RT to improve glycemic control and cardiometabolic health in T2D [26]. Research has also demonstrated that RT can improve cardiometabolic outcomes, such as increased insulin sensitivity and improved glycemic control, blood lipid profiles, and blood pressure (BP) in T2D [27–33]. RT is an effective intervention to manage T2D and offers a valid alternative to AT, which can be more difficult to perform in individuals with certain comorbidities associated with T2D, such as obesity, osteoarthritis, peripheral vascular disease, and other physical disabilities [33, 34]. However, the onset of T2D can occur 4 to 7 years before clinical diagnosis, a time period in which the hyperglycemia-associated complications of T2D can manifest [35]. Therefore, data supporting the implementation of RT at an earlier stage of prediabetes or metabolic syndrome before the onset of T2D could offset years of costs and complications. In addition, although both AT and RT increase insulin sensitivity, RT may offer an added benefit of an increase in skeletal muscle glucose disposal area [36].

Given that RT can reduce HbA1c, LDL, TG, and BP, and increase insulin sensitivity and HDL in T2D [27–32], the potential for RT to independently reduce the risk of T2D and CVD at an earlier stage should be further explored. To our knowledge, no existing review or meta-analysis has examined the effects of RT alone on cardiometabolic health in adults at risk for T2D. A meta-analysis conducted by Strasser et al. examined changes in cardiometabolic outcomes in those with metabolic syndrome and T2D and found decreased HbA1c levels [37]. However, 11 of the 13 studies included exclusively T2D participants, only 40 of the 513 participants across those studies had metabolic syndrome or prediabetes, and they were analyzed together with T2D participants. Given that Strasser et al.’s review was conducted 10 years ago and participants with metabolic syndrome and T2D were combined in analyses, a recent systematic review and meta-analysis investigating the effects of RT in those at risk for T2D is needed. Consequently, this is the first review to quantitatively assess the impact of RT exclusively in individuals at risk for T2D.

The purpose of this systematic review and meta-analysis was to determine the effects of RT on cardiometabolic risk factors in adults at risk for T2D. In addition, for this information to be implemented in daily practice for clinicians to utilize, this research also examined which characteristics of RT programs are correlated with improved cardiometabolic outcomes.

## Methods

### Protocol and Registration

This systematic review and meta-analysis was conducted using Preferred Reporting Items for Systematic Reviews and Meta-Analysis guidelines [38] and registered with PROSPERO (Registration ID CRD42019122217), an international prospective registry for systematic reviews [39].

### Eligibility Criteria

The criteria for inclusion of studies were as follows: (1) published randomized or nonrandomized controlled trials; (2) participants were adults over the age of 18 years; (3) participants were individuals with prediabetes or at risk for diabetes (e.g., insulin resistant or metabolic syndrome), but did not have diabetes; (4) the trial consisted of a group that participated in RT as an isolated intervention compared to a control group (CG); (5) trials with dietary interventions were included if the same dietary intervention was applied to the RT and CG; (6) the trial reported data for primary or secondary measurements of interest; and (7) the study had to be published in English, or reliably translatable using online processing [40].

### Information Sources and Search Strategy

To identify studies that examined the impact of RT on cardiometabolic outcomes in individuals with increased cardiometabolic risk, an electronic literature search was conducted on PubMed, Embase, Web of Science, and Cochrane databases from inception to December 2019 with no date restriction. Keywords and medical subject heading (MeSH) terms related to prediabetic states and RT were entered into the databases to identify studies that fit the criteria. The complete search strategy is reported (see Electronic Supplementary Material File 1).

### Study Selection, Data Collection, and Data Items

To determine eligibility criteria, the titles and abstracts were screened independently by two reviewers, and disagreements were resolved by consensus or a separate reviewer. Of the relevant studies, full texts were screened by the same two reviewers, and disagreements were resolved by consensus or a separate reviewer. Data extraction and quality assessment were conducted independently by two reviewers, and disagreements were resolved by a separate reviewer. Data were extracted from each individual study on the following study characteristics: participant demographics, RT intervention characteristics, pre- and post-intervention means, and standard deviations for outcome variables, study design, duration, year of publication, adverse events, criteria for prediabetes classification, presence of dietary interventions, data for risk for bias assessment, and number of dropouts.

### Risk of Bias

The risk of bias of included studies was assessed independently by two reviewers using the Cochrane Risk of Bias tool [41], and disagreements were resolved by a third reviewer. Publication bias was assessed by Egger’s asymmetry test [42].

### Summary Measures and Statistical Analyses

Primary measurements of interest were HbA1c, FPG, HDL, TG, waist circumference (WC), systolic blood pressure (SBP), and diastolic blood pressure (DBP). Secondary measurements of interest were body fat % (BF%), body mass index (BMI), fasting insulin, TC, LDL, and HOMA-IR, which is a measure of insulin resistance. If intention-to-treat analyses were reported in the studies, then those data were extracted from those studies [43, 44]. A meta-analysis was conducted to determine changes in adiposity, glycemic control, insulin resistance, blood lipids/lipoproteins, and BP following RT interventions. Meta-analyses were executed using Comprehensive Meta-Analysis (Biostat, V 2.2.064, Englewood, NJ, USA). Pooled data using a random-effects model were used to investigate differences between intervention and CG. Differences in means were calculated for each study, and a summary of overall difference in means recorded for each outcome measure. The *p*-value was set at < 0.05.

### Additional Analyses

For variables with sufficient numbers of included studies, and with high levels of heterogeneity, a further moderator analysis was performed using a mixed effects model.

## Results

### Study Selection and Study Characteristics

**Fig. 1:**
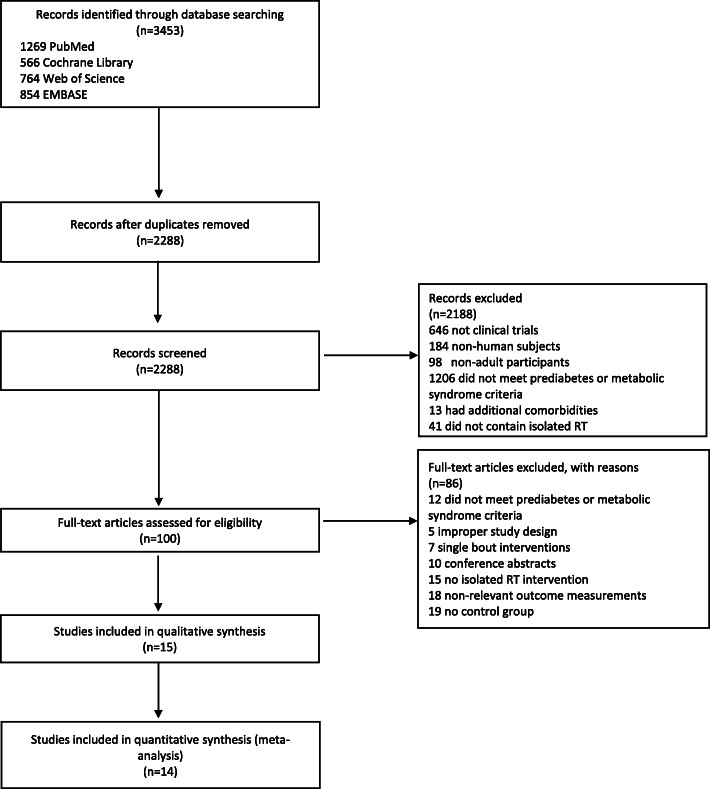
Flow chart for selection of studies for systematic review

The selection process for the studies included in this analysis is outlined in Fig. 1. From the 3453 references obtained from the electronic literature search, 1165 duplicates were removed. Of the remaining 2288 studies, 2188 were excluded by title and abstract. The full text of the remaining 100 studies was reviewed for potential inclusion. There were 15 articles that met the inclusion criteria, of which two used the same study participants [45, 46]. Ultimately, 14 trials with 668 participants at risk for T2D were included in this analysis.

### Study Characteristics

The characteristics of the 14 eligible studies are summarized in Table 1.

**Table 1:** Study characteristics

Study	Country	Number of participants	Age	Metabolic risk criteria	Duration (weeks)	Medications	Findings
Alvarez et al. [40]	Chile	CON=13 RT=8	CON= 40.1 ± 11.4 RT=33.9 ± 9.3	Elevated glucose	12	NR	↓HOMA-IR, FPG (*p*=0.011) with RT
Dai et al. [47]	China	CON=35 RT=31	CON=55–75 RT=55–75	Prediabetes	96	Lipid lowering (3 CON, 6 RT)	↓HbA1c, FPG (*p*<0.001), TC, LDL (*p*<0.001), TG (*p*=0.03), and ↑HDL (*p*<0.001) with RT
Devallance et al. [48]	USA	CON=16 RT=14	CON=51 ± 4 RT=51 ± 3	Metabolic syndrome	8	Antihypertensive (41% CON, 38% RT). Cholesterol lowering (19% CON, 23% RT)	No significant changes in outcomes of interest
Flández et al. [49]	Chile	CON=20 RT=20	CON=46.47 ± 3.71 RT= 46.47 ± 3.71	Metabolic syndrome	12	NR	↓HbA1c (*p*<0.05) with RT
Huffman et al. [50]	USA	CON=20 RT=20	CON=18–70 RT=18–70	Dyslipidemia	24	NR	No significant changes in outcomes of interest
Korkmaz et al. [45]; Venojärvi et al. [46]	Finland	CON=40 RT=36	CON=54 ± 1 RT=40–65	Impaired glucose regulation	12	Lipid lowering (25% CON, 22% RT). Antihypertensive (30% CON, 33% RT).	No significant changes in outcomes of interest
Levinger et al. [51]	Australia	CON=15 RT=15	CON=52.3 ± 5.8 RT=51.6 ± 7.1	Metabolic risk factors according to the International Diabetes Federation	10	Antihypertensives, cholesterol lowering, and metformin.	No significant changes in outcomes of interest
Levinger et al. [52]	Australia	CON=14 RT=15	CON=51.21 ± 7.33 RT=51 ± 7	Metabolic risk factors according to the International Diabetes Federation	10	Antihypertensives, cholesterol lowering, and metformin.	No significant changes in outcomes of interest
Mager et al. [53]	Finland	CON=18 RT=14	CON=60 ± 7 RT=60 ± 7	Impaired fasting glucose and 2 metabolic syndrome factors	33	NR	No significant changes in outcomes of interest
Marcus et al. [54]	USA	CON=6 RT=10	CON=53.2 ± 6.5 RT=56.3 ± 6.4	Impaired glucose tolerance	12	NR	No significant changes in outcomes of interest
Stensvold et al. [55]	Norway	CON=11 RT=11	CON=47.3 ± 10.2 RT=50.9 ± 7.6	Metabolic syndrome	12	Antihypertensive and lipid lowering. (4 CON, 8 RT).	↓WC with RT
Turri-Silva et al. [56]	Brazil	CON=12 RT=19	CON=51.21 ± 7.33 RT=51.42 ± 5.22	Metabolic syndrome	12	NR	No significant changes in outcomes of interest
Yuan et al. [44]	China	CON=83 RT=82	CON=60.73 ± 5.83 RT=59.91 ± 5.92	Prediabetes	24	Antihypertensives and cholesterol lowering.	↓HbA1c (*p*<0.001), BMI, SBP (*p*<0.05) with RT
Yan et al. [43]	China	CON=35 RT=35	CON=60.31 ± 7.56 RT=62.06 ± 8.11	Prediabetes	24	NR	↓FPG (*p*=0.004) with RT

*NR* not reported, *CON* control, *RT* resistance training, *HbA1c* glycosylated hemoglobin, *FPG* fasting plasma glucose, *TC* total cholesterol, *LDL* low-density lipoprotein, *HDL* high-density lipoprotein, *BMI* body mass index, *SBP* systolic blood pressure

All 14 studies were randomized [40, 43–55] or non-randomized controlled trials [56] published between 2008 and 2019. Thirteen studies were published in English [43–56] and one in Spanish [40]. The exercise interventions were conducted in health centers [40, 49], clinics [43–56], performance labs [48, 51, 52], training/fitness centers [45, 46], research centers [53, 54], and universities [55]. One study reported data values in medians and ranges [43] which were converted into mean and standard deviations using a conversion method [57]. Another study reported transformed means and standard deviations, and these values were back transformed [44]. Only two studies reported intention-to-treat analyses [43, 44].

### Participant Characteristics

The final analysis included 668 participants, adults aged ≥18 years. One study included only males [45, 46], three had only females [40, 49, 54], and the rest consisted of males and females [43, 44, 47, 48, 50–53, 55, 56]. To meet the criteria for at risk for T2D, participants met the criteria for metabolic syndrome in four studies [48, 49, 55, 56], impaired FPG or glucose tolerance in six studies [43–47, 53, 54], elevated BG (>100 mg/dL) in one study [40], dyslipidemia in one study [50], and ≥two metabolic risk factors according to the International Diabetes Federation in two studies [51, 52].

### RT Intervention Characteristics

The RT protocols of the individual studies are reported in Table 2.

**Table 2:** Resistance training protocol characteristics

Study	Intensity (% of 1RM unless otherwise indicated)	Repetitions per set (#)	Sets per exercise (#)	Exercises	Modality (elastic bands, machines, free weights, body weight)	Rest intervals	Frequency of training (sessions per week)	Design	Dietary component
Alvarez et al. [40]	1 min to failure	NR	3	Squat, biceps flexion and extension, ankle flexion and extension, shoulder flexion and extension, elbow flexion and extension.	Free weights	120 s between sets	2	Control, AT, RT, AT+RT	No
Dai et al. [47]	60–80%	NR	NR	Leg press, leg extension, chest press, pull downs, rowing, shoulder press	Elastic bands	NR	3	Control, AT, RT, AT+RT	Yes
Devallance et al. [48]	60–85%	8–12	3	Leg press, chest press, lat pull down, leg curl, shoulder press, leg extension.	Machines	NR	3	Control, RT. healthy and MetS	No
Flández et al. [49]	NR	10–15	3–4	Horizontal chest press on fitball, biceps curl, horizontal French press on fitball, military press sitting on fitball, vertical rowing, inclined rowing, reverse fly, front lunge, lateral lunge, half squat	Free weights (circuit)	30 s active rest between exercises. 60s between circuits	3–4	Control, RT (FW), RT (bands)	No
Huffman et al. [50]	NR	8–12	3 sets per day	Upper and lower body exercises	Machines	NR	3	CON, RT, AT (3 groups of varying intensity), AT/RT	No
Korkmaz et al. [45]; Venojärvi et al. [46]	50–85%	NR	NR	Leg press, bench press, leg extension, lateral pull-down, leg flexion, shoulder flexion, explosive leg squats, squat jumps, calf jumps, heel raises, pushups, abdominal flexion, back extension	Machines and free weights	NR	3	CON, RT, walking	No
Levinger et al. [51]	40–85%	8–20	2–3	Chest press, leg press, lateral pull-down, triceps pushdown, knee extension, seated row, biceps curl, abdominal curl	NR	NR	3	CON, RT. (high- and low-risk factor group for each)	No
Levinger et al. [52]	40–85%	8–20	2–3	Chest press, leg press, lateral pull-down, triceps pushdown, knee extension, seated row, biceps curl, abdominal curl	NR	NR	3	CON, RT. (high- and low-risk factor group for each)	No
Mager et al. [53]	60–70%	10–16	1–2	NR	NR	NR	2–4	CON, RT, AT, weight reduction	No
Marcus et al. [54]	Somewhat hard (RPE)	NR	NR	Eccentric ergometer	Ergometer	NR	3	CON, RT	Yes
Stensvold et al. [55]	80%	8–12	3	Low row, bench press, hack lift. Lateral raise, triceps pulldown, biceps curl, low row, plank.	NR	NR	3	CON, AT, RT, AT+RT	No
Turri-Silva et al. [56]	30–100%	2–20	1–2	Leg press, leg curl machine, extensor machine (leg). Biceps, triceps, pectoral, back (all machines)	Machines	40–90s	3	CON, RT (CRT), RT (FRT)	No
Yuan et al. [44]	60%	10–15	NR	Leg press, leg extension, chest press, pull down, row, calf raise, leg curl, shoulder press, straight arm forwards/backwards, leg rotation right/left, crunch	Bungee cord	NR	3	CON, AT, RT	Yes
Yan et al. [43]	60%	10–15	NR	Leg press, leg extension, chest press, pull down, row, calf raise, leg curl, shoulder press, straight arm forwards/backwards, leg rotation right/left, crunch	Bungee cord	NR	3	CON, AT, RT	Yes

*NR* not reported, *CON* control, *RT* resistance training, *AT* aerobic training, *CRT* conventional resistance training, *FRT* functional resistance training, *FW* free weights, *MetS* metabolic syndrome, *RPE* ratings of perceived exertion

The trials ranged from 8 to 96 weeks in duration, with frequencies of two to four sessions per week. With the exception of one trial [53], the exercise interventions in the remaining trials were supervised [40, 43–56]. With the exception of one study [54], all interventions using free weights, machines, or resistance bands incorporated multi-joint upper and lower extremity exercises. Of the seven studies for which total weekly number of sets could be calculated, four performed ≥52 sets [48, 49, 51, 52], and three performed <52 sets [40, 50, 55]. The compliance rates ranged from 67 to 100%.

### Risk of Bias Within and Across Studies

The risk of bias for included studies is summarized in Electronic Supplementary Material File 2. The method of randomization was described in six studies [43, 44, 47, 48, 55, 56]. Outcome assessors were blinded in two studies [47, 48]. One study had a dropout rate of greater than 20% in the RT group [45, 46]. There was no evidence of significant publication bias for HbA1c (*t*=1.232 95% confidence interval (CI) −9.035 to 23.446; *p*=0.143), FPG (*t*=1.234, 95% CI −5.149 to 16.401; *p*=0.128), TC (*t*=0.429, 95% CI −15.509 to 11.273; *p*=0.342), BF% (*t*=0.788, 95% CI −13.111 to 18.989; *p*=0.257), BMI (*t*=2.249, 95% CI −1.396 to 8.119; *p*=0.055), insulin (*t*=0.695, 95% CI −13.512 to 8.105; *p*=0.263), HOMA IR (*t*=0.614, 95% CI −31.267 to 23.453; *p*=0.301), HDL (*t*=1.146 95% CI −4.771 to 11.478; *p*=0.158), LDL (*t*=0.094, 95% CI −31.447 to 33.360; *p*=0.466), TG (*t*=0.219, 95% CI −6.885 to 5.756; *p*=0.417), SBP (*t*=0.775, 95% CI −13.995 to 24.832; *p*=0.241), or DBP (*t*=0.691, 95% CI −8.282 to 13.772; *p*=0.264). There was evidence of possible publication bias for WC (*t*=5.249, 95% CI 3.291 to 10.680; *p*=0.003). *P*-values were reported for the 1-tailed test.

### Syntheses of Results

#### Summary of Effect Sizes of RT

**Fig. 2:**
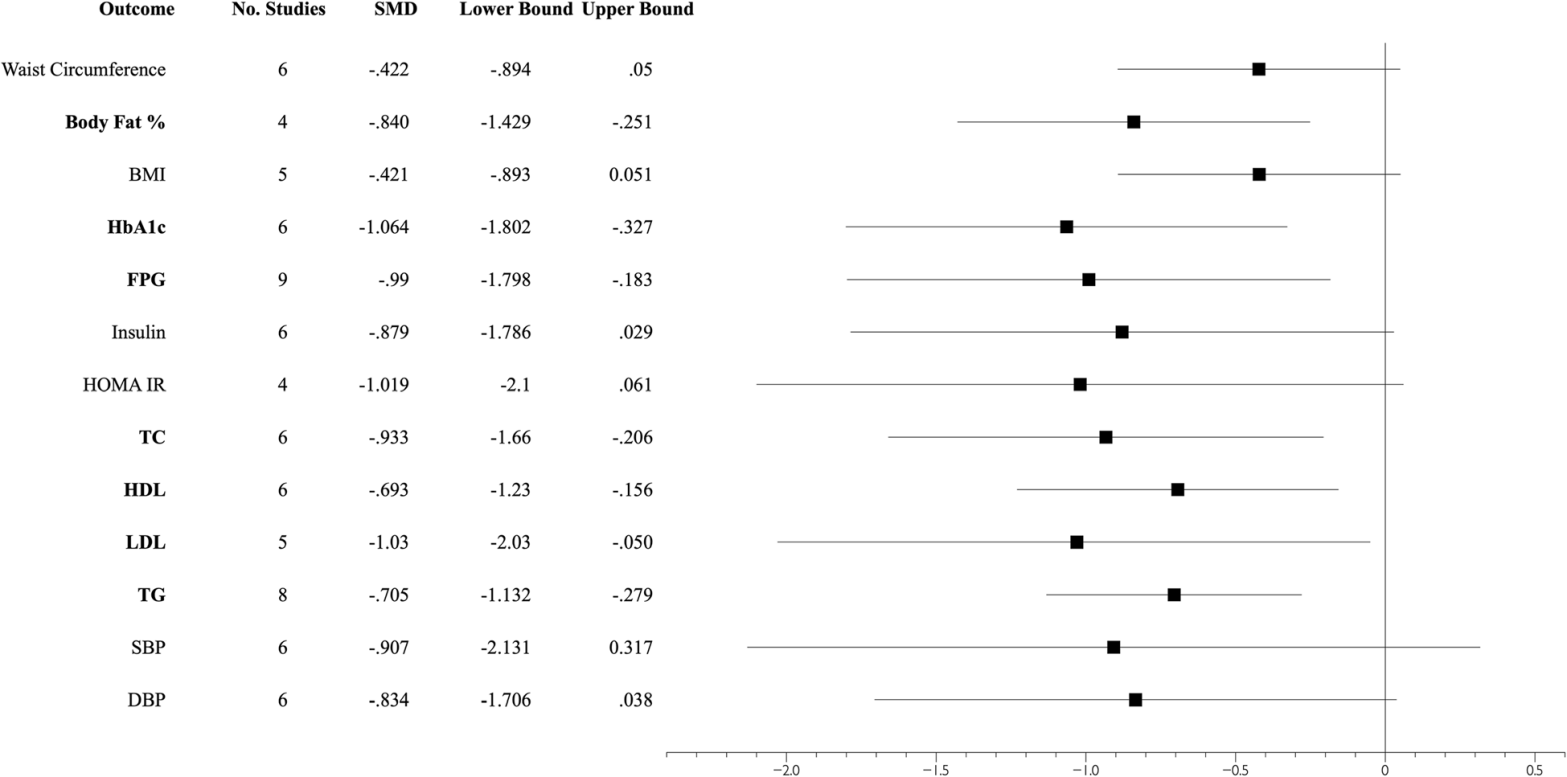
Forest plot for the outcome variables. Black squares with horizontal lines indicate the standardized mean difference (SMD) and 95% confidence interval between the intervention and control groups for outcome variables

Figure 2 shows the forest plot overall for the outcome variables. Individual forest plots for each outcome variable are included in Electronic Supplementary Material File 3. For RT compared to controls, significant differences were found for HbA1c (*p*=0.005), FPG (*p*=0.016), TC (*p*=0.012), BF% (*p*=0.005), HDL (*p*=0.011), LDL (*p*=0.039), and TG (*p*=0.001). No changes in BMI (*p*=0.081), HOMA-IR (*p*=0.064), SBP (*p*=0.146), DBP (*p*=0.061), fasting insulin (*p*=0.058), or WC (*p*=0.080) were found. The mean reduction for HbA1c, when assessed using mean difference, was 0.29% (95% CI −0.433 to −0.152; *p*=0.001).

#### Summary of RT Characteristics

**Fig. 3:**
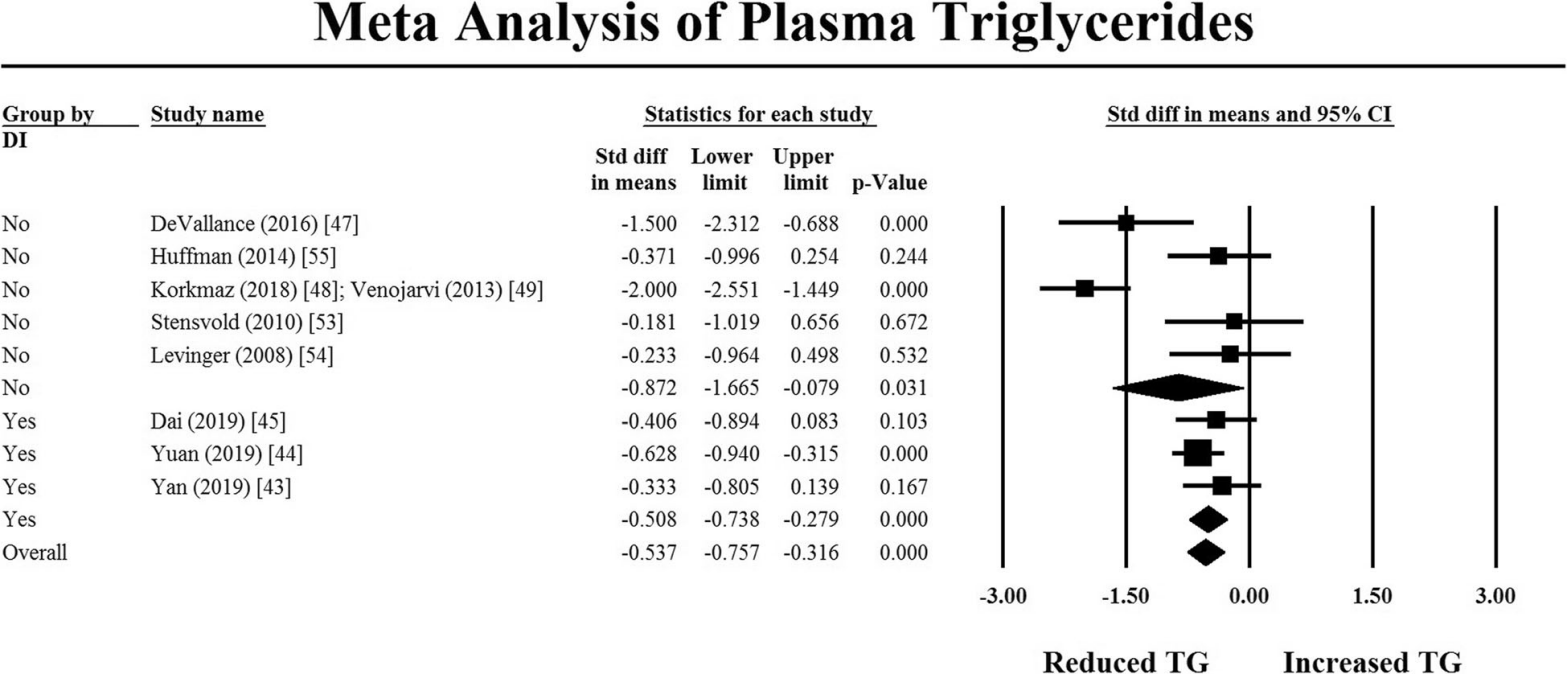
Moderator analysis on TG to compare studies which included a dietary component versus those that did not. Black-filled squares represent the mean and 95% confidence interval for individual studies. Open squares represent pooled mean and 95% confidence interval for subgroups. Filled diamond represents mean and 95% confidence interval for all pooled results

**Fig. 4:**
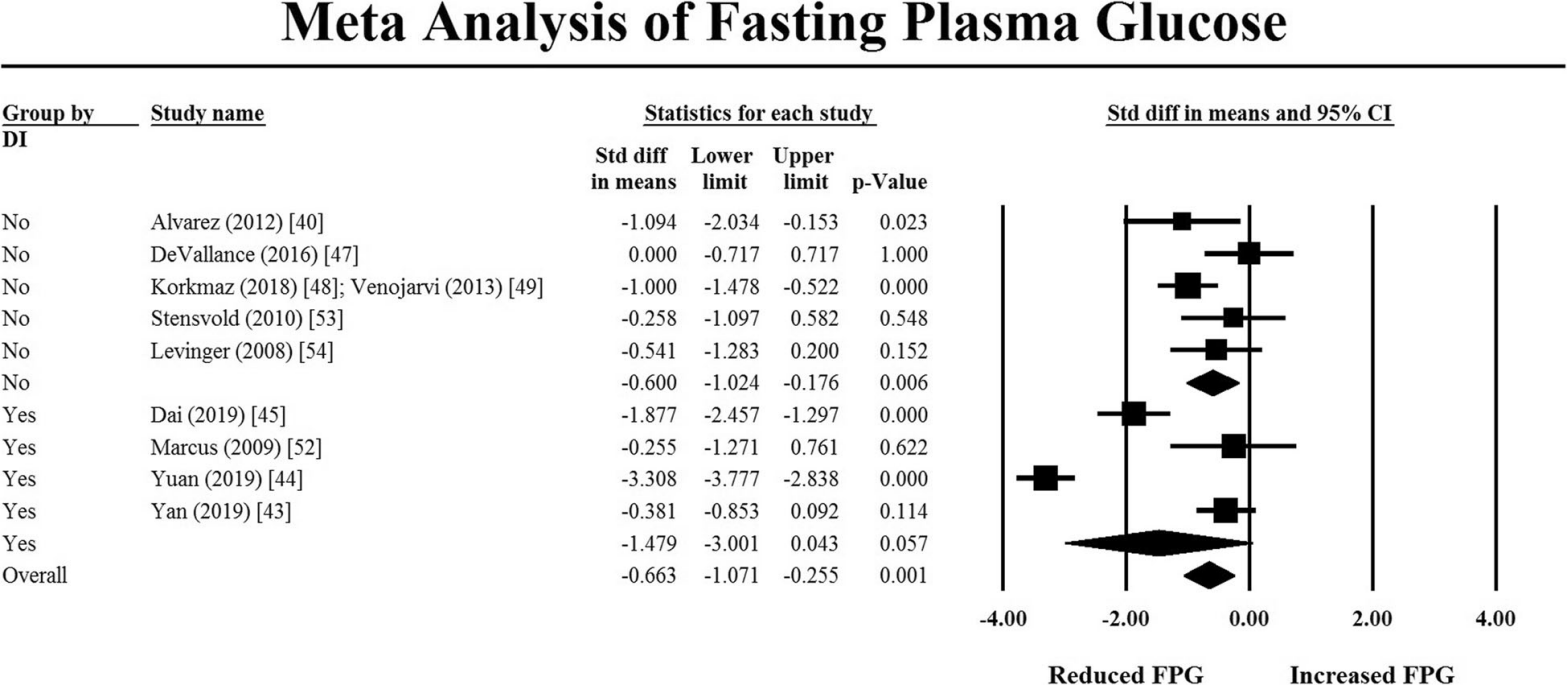
Moderator analysis on FPG to compare studies which included a dietary component versus those that did not. Black-filled squares represent the mean and 95% confidence interval for individual studies. Open squares represent pooled mean and 95% confidence interval for subgroups. Filled diamond represents mean and 95% confidence interval for all pooled results

A subsequent moderator analysis comparing the effect of RT with and without a dietary component on TG and FPG was undertaken using a mixed effects analysis. The results are depicted in the forest plots in Figs. 3 and 4. For FPG, there was no evidence that the addition of a dietary component increased the effectiveness of RT (*p* = 0.275). Similarly, for TG, there was also no difference in the effect size of studies with and without a dietary component (*p* = 0.388). When comparing intervention duration to the effect of the intervention on FPG and TG, there was no relationship between duration and the study effect size. Insufficient data reporting precluded further analyses of the effects of intervention characteristics on outcome variables.

## Discussion

This systematic review and meta-analysis evaluated the effectiveness of RT on cardiometabolic outcomes in individuals with increased cardiometabolic risk. The findings support the use of RT to prevent the onset of T2D. Improvements in glycemic control, adiposity, and blood lipids were evident. The addition of a dietary component to RT was not more effective in reducing FPG than RT alone, and intervention duration had no effect on FPG or TG.

### RT and Glycemic Control

This meta-analysis showed significant reductions in HbA1c and FPG in individuals with increased metabolic risk undergoing RT interventions alone compared to a CG, demonstrating the potential of RT to improve glycemic control and prevent hyperglycemia-associated complications. These results are consistent with a number of previous reports of RT decreasing HbA1c in those with T2D [27, 37, 58]. Given that greater reductions in HbA1c are seen in those with higher baseline HbA1c levels [59], the higher baseline HbA1c levels in previous reviews utilizing T2D populations would be expected to allow more scope for larger decreases in HbA1c. Comparatively, it was previously unknown if significant decreases in HbA1c would be seen in populations at risk for T2D, given their lower baseline HbA1c levels. The results of the present review demonstrate a significant improvement in glycemic control, despite lower baseline HbA1c values, suggesting that the incorporation of RT early after an “at risk” diagnosis may serve as an effective strategy to prevent the progression to T2D.

Of the three studies [44, 47, 49] in the present review that decreased HbA1c, one [49] utilized a circuit training protocol with free weights, consisting of ten consecutive exercises with 30 s of active recovery (e.g., jogging or mobility work) between exercises and 60 s of rest between circuits for a total of three to four circuits. Due to the aerobic aspect of circuit training [60], increased aerobic capacity could have impacted the results of this study, which may be reflected in the post-intervention 6-min walk distance increase among RT participants. However, these participants also demonstrated post-intervention increases in maximal voluntary isometric strength of the upper and lower limbs. Given that combined AT and RT has shown improvements in cardiometabolic outcomes [26], both the strength and aerobic aspects of this training protocol could have contributed to decreased HbA1c levels.

There are multiple potential mechanisms by which RT can improve glycemic control. The pathogenesis of impaired glucose regulation and eventually T2D is largely influenced by insulin resistance, with decreased insulin-stimulated glucose uptake in tissues resulting in elevated BG levels [61]. One potential mechanism involves skeletal muscle providing significant glucose uptake via glucose transporters [62] such as glucose transporter type 4 (GLUT4), which performs insulin-stimulated glucose uptake [63]. RT can increase the protein content of GLUT4, and increased muscle mass can enhance glucose uptake [64]. Therefore, characteristics of RT programs that enhance muscular hypertrophy may improve glycemic control. However, single bouts of RT have also shown decreased glucose levels in T2D [65], suggesting certain aspects of RT improve glycemic control independent of muscular hypertrophy.

An indirect mechanism by which RT can improve glycemic control is through increased basal metabolic rate, which aids in fat loss [66]. Decreased levels of adipose tissue can increase insulin sensitivity, as obesity is a contributing factor to peripheral insulin resistance and subsequent elevated BG levels [67, 68]. The present analysis showed significant decreases in BF% (*p*=0.005). However, only one study that demonstrated a decrease in HbA1c or FPG reported changes in BF% [40]. Thus, whether the changes in glycemic control were the result of changes in adiposity could not be determined. No significant changes in BMI were found in this analysis, which could be due to increased muscle mass offsetting changes in BMI from fat loss [69]. The use of RT to reduce fat mass and visceral adipose tissue has been reported in metabolic syndrome and obesity [37, 70]. Given that abdominal obesity/WC is a component of metabolic syndrome, decreasing abdominal obesity through RT could decrease metabolic risk. However, the present analysis showed no change in WC (*p*=0.080).

### RT and Blood Lipids

RT resulted in significant decreases in TC, LDL, and TG, and a significant increase in HDL levels. The results of the present analysis are in contrast to a previous review, which showed no significant differences in TC, LDL, TG, and HDL after RT in metabolic syndrome and T2D [37]. One reason for this difference could be differences in baseline values and participant characteristics. A majority of the participants from the previous review had T2D [37]. Another contribution to this difference could be that six [40, 45–49, 51] of the nine studies [40, 43, 45–51, 55] in the present review that assessed blood lipids used cholesterol-lowering drugs in some participants. The participants of the study [47] that showed significant changes in all blood lipid parameters (TC, LDL, TG, and HDL) had six individuals in the RT group and three individuals in the CG consistently utilizing lipid-lowering drugs (statins and ezetimibe). However, this study also concluded that medication use did not vary significantly between groups [47]. Therefore, RT in combination with pharmacologic therapy may be effective for blood lipid management in individuals at risk for T2D. However, without knowing how much medication use influenced the change, these results are inconclusive. Also, this same study was 96 weeks in duration [47]. The previous review included two studies lasting longer than a year that reported blood lipid values, and one reported significant increases in HDL, and significant decreases in LDL and TG [37]. It is possible, therefore, that longer interventions are required to achieve meaningful improvements in lipid profiles. However, controlled trials of 6 and 14 weeks have shown benefits of RT in LDL and TG in healthy adults, so duration of intervention may not be the determining factor [71, 72]. Regarding the use of RT in improving lipid profile, a separate meta-analysis found significant changes in TC, LDL, and TG following RT in healthy adults [73]. However, further studies in populations with increased metabolic risk are required to quantify the benefits of RT on blood lipids in populations at risk for T2D. Based on the current literature and the findings of the present analysis, it appears that, at minimum, in combination with other lifestyle modifications, RT can be beneficial for improving blood lipid profiles and subsequent risk of coronary artery disease in adults with increased cardiometabolic risk.

### RT and BP

The lack of change in SBP in the present review is in contrast to a previous review that showed a significant reduction in SBP in those with metabolic syndrome [74]. However, three of the seven studies in the previous review included T2D populations, one utilized a combined AT and RT intervention, and two incorporated weight loss into the interventions. The combination of the significant impact of weight loss on BP [75], AT on BP [76], and higher rates of elevated BP in T2D populations at baseline [77] allowing for greater decreases in BP [78] could have all contributed to this difference. Significant reductions in SBP have also been shown in individuals with prehypertension, hypertension, and normal BP [79, 80]. The lack of significant change in SBP in the present review could be due to the fact that every study [40, 43, 45, 46, 48, 52, 55] that reported changes in SBP had lower baseline SBP in the RT group compared to the control, allowing for less overall decrease [78]. In addition, differences in antihypertensive drug use between the control and RT group could have influenced results, as six studies [44–46, 48, 51, 52, 55] used antihypertensive drugs in both the control and RT groups.

### RT and Insulin

The non-significant changes in insulin levels can possibly be explained by the pathophysiology and progression of an insulin-resistant state. There is a compensatory increase in insulin secretion before glucose abnormalities develop in T2D [81]. Since the studies in the present review only included participants at risk for T2D, these individuals may still be in the stages of a compensatory increase in insulin secretion, or they may have had a lack of elevated insulin levels at baseline.

### RT Program Characteristics

The moderator analysis on dietary intervention suggested that studies including dietary components did not result in significantly lower reduction in TG or FPG than those that did not. However, of the four studies that reported dietary interventions in the CG and RT group [43, 44, 47, 54], one asked participants to follow a weight maintenance diet with specific macronutrient ratios after a session with a dietitian [47], one provided a diet handout and only recorded the same diet 3 days prior to pretraining testing and posttraining testing [54], and two studies reported strict dietary interventions with individualized meal plans to meet macronutrient ratios and all food intake recorded [43, 44]. It is possible that more strict control of diet could impact these results. The results of the meta-regression on duration of exercise affecting FPG and TG did not show a greater effect with longer studies. Taken together, these findings suggest that the effect of RT is greater than dietary advice or duration. However, there were fewer long-term studies, as only five [43, 44, 47, 50, 53] of the 14 studies [40, 43–49, 51–56] were longer than 12 weeks. Also, the most significant decreases in FPG and TG may be seen earlier in interventions, with the level of decrease attenuating over time and reaching a plateau [82, 83]. More information is needed on the interaction between RT, dietary interventions, and intervention duration.

There were not enough studies reporting exercise intensity to conduct a meta-regression on the impact of training intensity on glycemic outcomes. Of the three studies [44, 47, 49] in the present review that decreased HbA1c, two [44, 47] reported intensity in terms of one-repetition maximum (1-RM), utilizing a minimum intensity of 60% 1-RM, and one study [49] reported intensity in terms of the OMNI-Resistance Exercise Scale (OMNI-RES), using a range of 7–9, interchangeable with a rating of perceived exertion (RPE) of 15–18 on Borg’s RPE scale of 6–20 [84]. Two studies reported repetitions per set and utilized 10–15 repetitions per set [44, 49]. A previous meta-analysis reported greater decreases in HbA1c in T2D in those undergoing higher intensity RT compared to lower intensity RT, but acknowledged those changes were possibly impacted by different baseline HbA1c levels [58]. Additionally, another meta-analysis demonstrated that higher intensity (75–100% 1RM) RT in T2D produced greater decreases in HbA1c than lower intensity (20–75% 1RM) RT [85]. The results of the present analysis provide qualified support for these previous findings, given that studies reporting decreased HbA1c all utilized intensities of 60% 1-RM or greater, and thus the recommendation for incorporation of higher load training in individuals with prediabetes.

It is important to note that recent studies have suggested similar potential for hypertrophy from lower load training, as low as 30% of 1-RM, in healthy populations [86]. However, lower load training requires training significantly closer to repetition failure compared to higher load training to induce comparable levels of hypertrophy [86]. Further studies are needed that examine training to failure with lower intensities in those with impaired glucose regulation in order for recommendations of intensities below 60% 1-RM to be implemented in this population. The summary for RT recommendations for individuals at metabolic risk is reported in Table 3.

**Table 3:** Summary of recommendations for RT in adults at risk for T2D

Component	Guidelines
**Intensity and repetitions**	A range of 10–15 repetitions at intensities above 60% 1-RM may reduce HbA1c levels [44, 47, 49] and improve lipid profile [44]. All sets should be performed with an RPE of 15–18 according to Borg’s RPE scale (within one to three repetitions from failure) [49, 83, 87].
**Exercise modality**	Resistance band [44, 47] and free weight [49] training are both RT modalities that are effective for improving glycemic control and lipid profile.
**Exercise selection**	Multi-joint (e.g., leg press, bench press, lat pulldown, row, shoulder press) and single joint (e.g., leg extension, biceps curl, triceps pushdown) exercises are acceptable, with the majority of exercises being multi-joint exercises.
**Frequency**	A minimum of three sessions per week should be performed.

*RT* resistance training, *RPE* ratings of perceived exertion

These recommendations were based on the findings of the present analysis, previous reviews, and the joint statement by the American College of Sports Medicine and American Diabetes Association on RT in T2D [88]. The 2016 resistance training guidelines of the American Diabetes Association advised RT 2 to 3 days per week, at 10–15 repetitions per set for beginners and progressing to eight to ten repetitions per set in individuals with T2D [89]. Based on the present results and estimates of repetitions per set based on intensity, a range of 10–15 repetitions may be effective for improving glycemic control in individuals at risk for T2D, with multi-joint exercises prescribed at the lower end and single-joint exercises at the higher end of the range. The suggestion of higher vs. lower end of this range can be personalized towards individual needs. Lower loads may be preferred in elderly populations, leading to increased adherence [90]. Lower loads may also be preferred in those with or recovering from injuries, as they are associated with decreased training-related injuries compared to higher loads [91]. In addition, the recommendation for repetitions and intensity should be specific to exercise modality or selection. For example, utilizing higher repetitions with resistance bands may be more appropriate, due to the increased requirements of stability and ancillary muscular involvement of resistance bands [92–94]. Given that the studies [44, 47, 49] showing significant decreases in HbA1c or FPG utilized both free weights [47] and resistance bands [44, 47, 49], the recommendation for RT modality should be based on what is sustainable or feasible for individual needs, as long as sufficient tension and muscular fatigue are reached. RT with machines has also shown improved glycemic control in T2D [95, 96]. However, of the four studies in the present review that only used machines [48, 50, 54, 56], two did not measure outcomes of glycemic control [50, 56], and one only performed a knee extensor exercise [54]. The study in the present review that only performed a knee extensor exercise was the only study that did not incorporate multiple muscle groups and required participants to apply force with their feet to slow the backward rotating pedals of an ergometer to create eccentric muscle contractions of the lower extremity extensors [54]. This same study showed no decrease in FPG. Each set should meet an RPE of 15–18 according to Borg’s RPE scale which equates to training within one to three repetitions from failure [49, 84, 87]. Further studies specifying RT program characteristics in metabolic risk are needed to more specifically quantify these recommendations.

### Limitations

There are some limitations to this review that need to be acknowledged. One study included one participant with type 1 diabetes in the CG and one participant with type 1 diabetes in the training group [51], and one study included a single participant with T2D in the training group [52]. We elected to include the studies since the overall sample size was 15 in each group and the effect on the main outcome due to this addition was likely minimal. Nevertheless, future work should seek to ensure that the study cohorts meet the inclusion criteria without exception. Participants in seven studies [44–48, 51, 52, 55] were using various BP and lipid-lowering medications in the control and RT group, which could have impacted results. Future studies should strive to control for medication usage to ensure that changes in the variables of interest are due to the resistance training intervention independent of pharmaceutical effects. The methods of randomization were not described in eight [40, 45, 46, 49–54] studies, contributing towards potential bias. One study had a dropout rate of 26.5% in the RT group and 17.5% in the CG, potentially influencing intervention response [45, 46]. There was evidence of possible publication bias for WC (*p*=0.003); however, there were only four studies in this comparison, so additional studies in the future are required for confirmation of this finding. Additionally, the reporting of RT intervention characteristics was variable, as factors such as rest time, modality, volume, effort, and changes in strength and cardiorespiratory fitness were not reported in several studies, and differences in training effort could have impacted results. Future studies should aim to quantify all aspects of RT interventions such as volume, intensity, and rest times so that a meta-regression can be conducted on these values. While HOMA-IR (*p*=0.064) [40, 43–46, 55], DBP (*p*=0.061) [40, 43–46, 48, 55], BMI (*p*=0.081) [40, 43, 44, 53, 55], and fasting insulin (*p*=0.058) [40, 43–46, 53, 54] had *p*-values close to 0.05, the lack of statistical significance may have been due to a lack of studies and/or lower sample sizes in the studies that investigated these variables. More studies should investigate the effects of resistance training on HOMA-IR, BP, and BMI, and ensure that sample sizes are sufficient for detecting a significant change. Finally, dietary intervention was not well controlled as only two [43, 44] of the four studies [43, 44, 47, 54] reporting dietary interventions mentioned strict dietary monitoring. Future studies incorporating diet should apply more controlled protocols so that the impact of dietary intervention can be more accurately determined.

## Conclusions

RT can reduce HbA1c and FPG in individuals at risk for developing T2D. Thus, RT may be an effective intervention for delaying or preventing the onset of T2D and can be recommended by clinicians to those at risk for T2D to improve cardiometabolic outcomes. Although the findings also suggest that RT may improve blood lipid profiles, and that a dietary component combined with RT did not result in larger reductions in FPG and TG than RT alone, more studies in the future are needed to confirm these findings.

## Supplementary Information

**Additional file 1: Electronic Supplementary Material File 1.** Search strategy.

**Additional file 2: Electronic Supplementary File 2.** Risk of bias table.

**Additional file 3: Electronic Supplementary File 3.** Forest plots for outcome variables. Black filled squares represent the mean and 95% confidence interval for individual studies. Filled diamond represents mean and 95% confidence interval for all pooled results.

## Data Availability

Data supporting the findings of this study are available from the corresponding author on request.

## Abbreviations

T2D: Type 2 diabetes
CVD: Cardiovascular disease
BG: Blood glucose
HbA1c: Glycosylated hemoglobin
FPG: Fasting plasma glucose
TC: Total cholesterol
LDL: Low-density lipoprotein
HDL: High-density lipoprotein
TG: Triglycerides
AT: Aerobic training
RT: Resistance training
BP: Blood pressure
CG: Control group
WC: Waist circumference
SBP: Systolic blood pressure
DBP: Diastolic blood pressure
BF%: Body fat %
BMI: Body mass index
SMD: Standardized mean difference
GLUT 4: Glucose transporter type 4
1-RM: One-repetition maximum
RPE: Rating of perceived exertion
