# Supplementary material for: Effectiveness of Resistance Training and Associated Program Characteristics in Patients at Risk for Type 2 Diabetes: a Systematic Review and Meta-analysis
Source: Sports Med Open. 2021 May 29;7:38. doi: 10.1186/s40798-021-00321-x (PMC8164651; doi:10.1186/s40798-021-00321-x)
Supplement: Supplementary file 1 — Additional file 1: Electronic Supplementary Material File 1. Search strategy. [file 40798_2021_321_MOESM1_ESM.pdf]

Article Title: Effectiveness of Resistance Training and Associated Program Characteristics in Patients at Risk for Type 2 Diabetes: a Systematic Review and Meta-Analysis.

Journal name: Sports Medicine

Authors: Raza Qadir[1](corresponding author), Nicholas F. Sculthorpe[2], PhD, Taylor Todd[3], Elise C. Brown[3], PhD

1. Oakland University William Beaumont School of Medicine

   586 Pioneer Dr,

   Rochester, MI 48309, USA

   Email: razaqadir@oakland.edu

2. University of the West of Scotland

   Lanarkshire, United Kingdom

3. School of Health Sciences

   Oakland University

   Rochester, MI 48309, USA

Electronic Supplementary Material File 1 Search strategy

Pubmed

- **Search entry-** ("Prediabetic State"[Mesh] OR prediabetes (tiab) OR hyperglycemia (tiab) OR "impaired fasting glucose" (tiab) OR "impaired glucose tolerance" (tiab) OR "glucose intoleran*" (tiab) OR "insulin resistan*" (tiab) OR "impaired plasma glucose" (tiab) OR "metabolic syndrome" (tiab) OR dysglycemia (tiab) OR "impaired glucose regulation" (tiab) OR "insulin insensitiv*" (tiab) OR "insulin resistan*") AND ("Resistance Training"[Mesh] OR "resistance training" OR "strength training" OR "resistance exercise" OR weightlifting OR weight-lifting OR "weight training" OR "weight bearing" OR "strength exercise" OR "strength* program")

Cochrane

- **Search entry-** Under title/abstract/keywords- ("Prediabetic State" OR prediabetes OR hyperglycemia OR "impaired fasting glucose" OR "impaired glucose tolerance" OR "glucose intolerant" OR "insulin resistant" OR "insulin resistance" OR "impaired plasma glucose" OR "metabolic syndrome" OR dysglycemia OR "impaired glucose regulation" OR "insulin insensitive") AND ("Resistance Training" OR "strength training" OR "resistance exercise" OR weightlifting OR weight-lifting OR "weight training" OR "weight bearing" OR "strength exercise" OR "strength program")

Web of Science

- **Search entry-** ("Prediabetic State"[Mesh] OR prediabetes (tiab) OR hyperglycemia (tiab) OR "impaired fasting glucose" (tiab) OR "impaired glucose tolerance" (tiab) OR "glucose intoleran*" (tiab) OR "insulin resistan*" (tiab) OR "impaired plasma glucose" (tiab) OR "metabolic syndrome" (tiab) OR dysglycemia (tiab) OR "impaired glucose regulation" (tiab) OR "insulin insensitiv*" OR "insulin resistan*") AND ("Resistance Training"[Mesh] OR "resistance training" OR "strength training" OR "resistance exercise" OR weightlifting OR weight-lifting OR "weight training" OR "weight bearing" OR "strength exercise" OR "strength* program")

Embase

- **Search entry-** ('prediabetic state':ti,ab,kw OR 'prediabetes':ti,ab,kw OR 'hyperglycemia':ti,ab,kw OR 'impaired fasting glucose':ti,ab,kw OR 'impaired glucose tolerance':ti,ab,kw OR 'glucose intolerance':ti,ab,kw OR 'glucose intolerant':ti,ab,kw OR 'insulin resistant':ti,ab,kw OR 'insulin resistance':ti,ab,kw OR 'impaired plasma glucose':ti,ab,kw OR 'metabolic syndrome':ti,ab,kw OR 'dysglycemia':ti,ab,kw OR 'impaired glucose regulation':ti,ab,kw OR 'insulin insensitive':ti,ab,kw OR 'insulin insensitivity':ti,ab,kw) AND ('resistance training':ti,ab,kw OR 'strength training':ti,ab,kw OR 'resistance exercise':ti,ab,kw OR 'weightlifting':ti,ab,kw OR 'weight-lifting':ti,ab,kw OR 'weight training':ti,ab,kw OR 'weight bearing':ti,ab,kw OR 'strength exercise':ti,ab,kw OR 'strength program':ti,ab,kw OR 'strengthening program':ti,ab,kw)

Declarations

Ethics approval and consent to participate Not applicable

Consent for publication Not applicable

Funding This study was supported through the Oakland University School of Health Sciences grant.

Competing interests Raza Qadir, Nicholas F. Sculthorpe, Taylor Todd, and Elise C. Brown declare that they have no competing interests.

Availability of data and material Data supporting the findings of this study are available from the corresponding author on request.

Author Contributions ECB and RQ designed the research and conducted the searches and screening. RQ and TT extracted the data, which were verified by ECB. NFS performed the statistical analyses. RQ wrote the manuscript with critical input from ECB and NFS.

Acknowledgements Not applicable.
