# Supplementary material for: Effectiveness of Resistance Training and Associated Program Characteristics in Patients at Risk for Type 2 Diabetes: a Systematic Review and Meta-analysis
Source: Sports Med Open. 2021 May 29;7:38. doi: 10.1186/s40798-021-00321-x (PMC8164651; doi:10.1186/s40798-021-00321-x)
Supplement: Supplementary file 2 — Additional file 2: Electronic Supplementary File 2. Risk of bias table. [file 40798_2021_321_MOESM2_ESM.pdf]

Article Title: Effectiveness of Resistance Training and Associated Program Characteristics in Patients at Risk for Type 2 Diabetes: a Systematic Review and Meta-Analysis.

Journal name: Sports Medicine

Authors: Raza Qadir[1](corresponding author), Nicholas F. Sculthorpe[2], PhD, Taylor Todd[3], Elise C. Brown[3], PhD

1. Oakland University William Beaumont School of Medicine

   586 Pioneer Dr,

   Rochester, MI 48309, USA

   Email: razaqadir@oakland.edu

2. University of the West of Scotland

   Lanarkshire, United Kingdom

3. School of Health Sciences

   Oakland University

   Rochester, MI 48309, USA

Electronic Supplementary File 2 Risk of bias table

Study	Treatment Allocation Concealed	Adequate Randomization	Participants Blinded	Outcome Assesors Blinded	Drop out rate at endpoint ≤20% or lower of number allocated to treatment	High adherence to intervention protocols for each treatment group?
Alvarez, 2012	NR	NR	No	NR	NR	Yes
Dai, 2019	Yes	Yes	No	Yes	Yes	Yes
DeVallance, 2016	Yes	Yes	No	Yes	Yes	Yes
Flandez, 2017	NR	NR	No	NR	NR	NR
Huffman, 2014	NR	NR	No	NR	NR	NR
Korkmaz, 2018	NR	NR	No	NR	No	No
Levinger, 2007	NR	NR	No	NR	Yes	Yes
Levinger, 2008	NR	NR	No	NR	Yes	Yes
Mager, 2008	NR	NR	No	NR	NR	NR
Marcus, 2009	NR	NR	No	NR	Yes	Yes
Stensvold, 2010	Yes	Yes	No	NR	Yes	Yes
Turri-Silva, 2018	NR	Yes	No	NR	Yes	Yes
Venojarvi, 2013	NR	NR	No	NR	No	No
Yuan, 2019	NR	Yes	No	NR	Yes	NR
Yan, 2019	No	Yes	No	NR	Yes	NR

NR= not reported

Declarations

Ethics approval and consent to participate Not applicable

Consent for publication Not applicable

Funding This study was supported through the Oakland University School of Health Sciences grant.

Competing interests Raza Qadir, Nicholas F. Sculthorpe, Taylor Todd, and Elise C. Brown declare that they have no competing interests.

Availability of data and material Data supporting the findings of this study are available from the corresponding author on request.

Author Contributions ECB and RQ designed the research and conducted the searches and screening. RQ and TT extracted the data, which were verified by ECB. NFS performed the statistical analyses. RQ wrote the manuscript with critical input from ECB and NFS.

Acknowledgements Not applicable.
