# Supplementary material for: Effectiveness of Resistance Training and Associated Program Characteristics in Patients at Risk for Type 2 Diabetes: a Systematic Review and Meta-analysis
Source: Sports Med Open. 2021 May 29;7:38. doi: 10.1186/s40798-021-00321-x (PMC8164651; doi:10.1186/s40798-021-00321-x)
Supplement: Supplementary file 3 — Additional file 3: Electronic Supplementary File 3. Forest plots for outcome variables. Black filled squares represent the mean and 95% confidence interval for individual studies. Filled diamond represents mean and 95% confidence interval for all pooled results. [file 40798_2021_321_MOESM3_ESM.pdf]

Article Title: Effectiveness of Resistance Training and Associated Program Characteristics in Patients at Risk for Type 2 Diabetes: a Systematic Review and Meta-Analysis.

Journal name: Sports Medicine

Authors: Raza Qadir[1](corresponding author), Nicholas F. Sculthorpe[2], PhD, Taylor Todd[3], Elise C. Brown[3], PhD

1. Oakland University William Beaumont School of Medicine

   586 Pioneer Dr,

   Rochester, MI 48309, USA

   Email: razaqadir@oakland.edu

2. University of the West of Scotland

   Lanarkshire, United Kingdom

3. School of Health Sciences

   Oakland University

   Rochester, MI 48309, USA

Electronic Supplementary File 3 Forest plots for outcome variables. Black filled squares represent the mean and 95% confidence interval for individual studies. Filled diamond represents mean and 95% confidence interval for all pooled results

Percentage Body Fat Changes

Study name	Statistics for each study						
	Std diff in means	Standard error	Variance	Lower limit	Upper limit	Z-Value	p-Value
Alvarez (2012)	-0.192	0.450	0.203	-1.075	0.691	-0.426	0.670
DeVallance (2016)	-1.500	0.414	0.171	-2.312	-0.688	-3.623	0.000
Korkmaz (2018); Venojarvi (2013)	-1.197	0.249	0.062	-1.686	-0.708	-4.799	0.000
Levinger (2007)	-0.340	0.368	0.135	-1.061	0.380	-0.925	0.355
	-0.840	0.301	0.090	-1.429	-0.251	-2.797	0.005

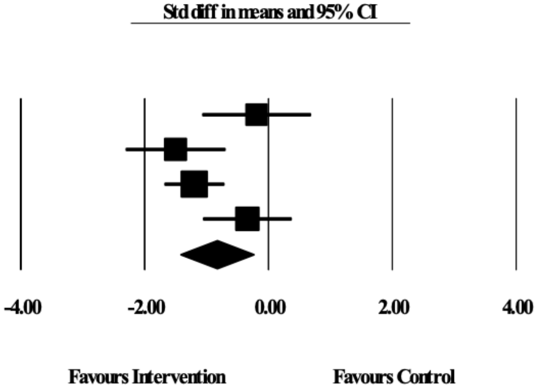

BMI Changes

Study name	Std diff in means	Standard error	Variance	Lower limit	Upper limit	Z-Value	p-Value
Alvarez (2012)	-0.219	0.451	0.203	-1.102	0.664	-0.486	0.627
Mager (2008)	-0.303	0.358	0.128	-1.005	0.400	-0.845	0.398
Stensvold (2010)	-0.046	0.426	0.182	-0.882	0.789	-0.109	0.913
Yuan (2019)	-1.035	0.166	0.027	-1.360	-0.710	-6.243	0.000
Yan (2019)	-0.187	0.240	0.057	-0.656	0.283	-0.779	0.436
	-0.421	0.241	0.058	-0.893	0.051	-1.746	0.081

Statistics for each study

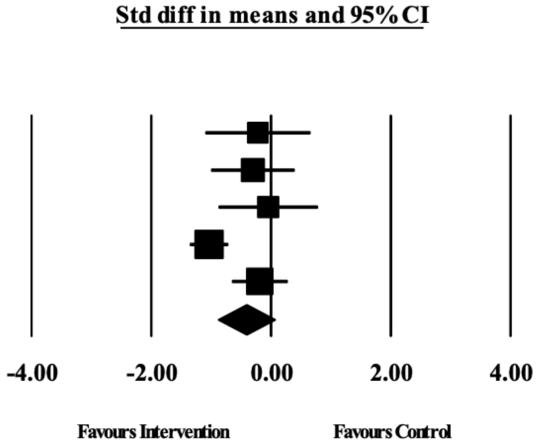

HbA1c Changes

Study name	Std diff in means	Standard error	Variance	Lower limit	Upper limit	Z-Value	p-Value
Dai (2019)	-1.659	0.286	0.082	-2.219	-1.099	-5.805	0.000
Flandez (2017)	-0.883	0.331	0.110	-1.532	-0.233	-2.665	0.008
Korkmaz (2018); Venojarvi (2013)	-1.000	0.244	0.059	-1.478	-0.522	-4.105	0.000
Stensvold (2010)	-0.187	0.427	0.183	-1.024	0.651	-0.436	0.663
Yuan (2019)	-2.315	0.201	0.040	-2.709	-1.920	-11.505	0.000
Yan (2019)	-0.207	0.240	0.057	-0.677	0.263	-0.863	0.388
	-1.064	0.376	0.141	-1.802	-0.327	-2.830	0.005

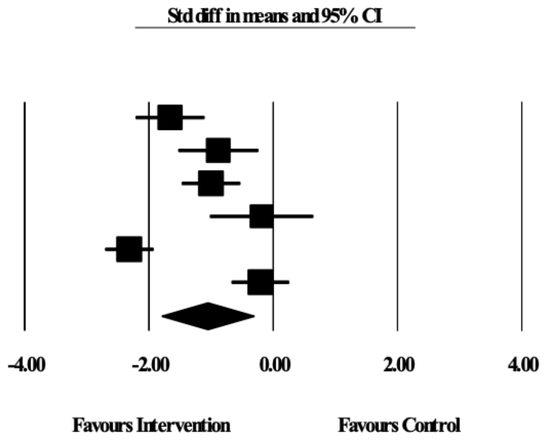

HDL Changes

Study name	Std diff in means	Standard error	Variance	Lower limit	Upper limit	Z-Value	p-Value
Dai (2019)	-0.707	0.254	0.065	-1.205	-0.209	-2.781	0.005
DeVallance (2016)	-1.000	0.388	0.151	-1.761	-0.239	-2.577	0.010
Stensvold (2010)	-0.085	0.427	0.182	-0.921	0.751	-0.200	0.842
Levinger (2008)	-0.750	0.384	0.148	-1.503	0.003	-1.951	0.051
Yuan (2019)	-1.457	0.175	0.031	-1.800	-1.114	-8.319	0.000
Yan (2019)	-0.030	0.239	0.057	-0.499	0.438	-0.127	0.899
	-0.693	0.274	0.075	-1.230	-0.156	-2.530	0.011

Statistics for each study

Std diff in means and 95% CI

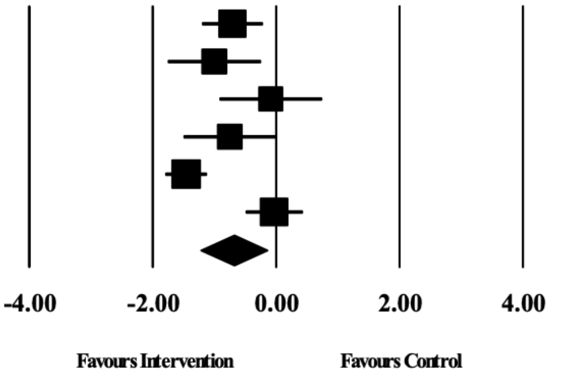

HOMA IR Changes

Study name	Std diff in means	Standard error	Variance	Lower limit	Upper limit	Z-Value	p-Value
Alvarez (2012)	-0.397	0.454	0.206	-1.286	0.492	-0.875	0.382
Korkmaz (2018); Venojarvi (2013)	-2.948	0.332	0.110	-3.598	-2.298	-8.891	0.000
Yuan (2019)	-0.566	0.159	0.025	-0.877	-0.255	-3.566	0.000
Yan (2019)	-0.192	0.240	0.057	-0.661	0.278	-0.800	0.423
	-1.019	0.551	0.304	-2.100	0.061	-1.849	0.064

Statistics for each study

Std diff in means and 95% CI

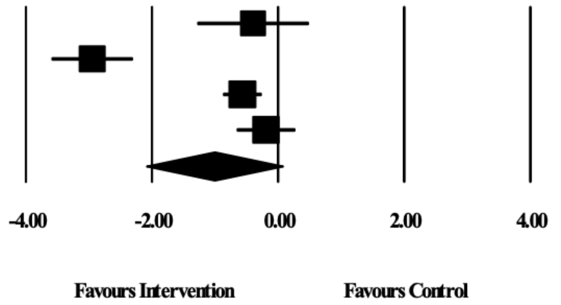

Plasma Insulin Changes

Study name	Std diff in means	Standard error	Variance	Lower limit	Upper limit	Z-Value	p-Value
	Statistics for each study						
Alvarez (2012)	-0.205	0.450	0.203	-1.088	0.678	-0.455	0.649
Korkmaz (2018); Venojarvi (2013)	-3.584	0.371	0.137	-4.310	-2.858	-9.673	0.000
Mager (2008)	-0.440	0.361	0.130	-1.146	0.267	-1.219	0.223
Marcus (2009)	-0.244	0.518	0.269	-1.259	0.772	-0.470	0.638
Yuan (2019)	-0.339	0.157	0.025	-0.647	-0.032	-2.163	0.031
Yan (2019)	-0.451	0.242	0.059	-0.925	0.024	-1.861	0.063
	-0.879	0.463	0.215	-1.786	0.029	-1.897	0.058

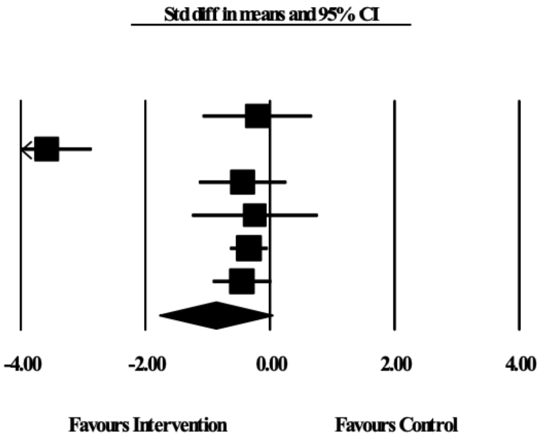

LDL Changes

Study name	Std diff in means	Standard error	Variance	Lower limit	Upper limit	Z-Value	p-Value
Dai (2019)	-0.453	0.250	0.062	-0.942	0.037	-1.812	0.070
Flandez (2017)	-0.105	0.316	0.100	-0.725	0.515	-0.333	0.739
Korkmaz (2018); Venojarvi (2013)	-3.000	0.335	0.112	-3.656	-2.344	-8.965	0.000
Yuan (2019)	-1.636	0.180	0.032	-1.988	-1.283	-9.095	0.000
Yan (2019)	0.000	0.239	0.057	-0.469	0.469	0.000	1.000
	-1.031	0.501	0.251	-2.013	-0.050	-2.059	0.039

Statistics for each study

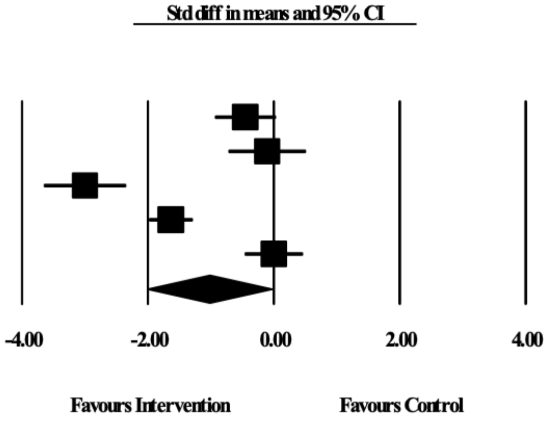

SBP Changes

Study name	Std diff in means	Standard error	Variance	Lower limit	Upper limit	Z-Value	p-Value
Alvarez (2012)	-0.400	0.454	0.206	-1.289	0.489	-0.883	0.377
DeVallance (2016)	-1.333	0.404	0.164	-2.126	-0.541	-3.297	0.001
Korkmaz (2018); Venojarvi (2013)	-0.060	0.230	0.053	-0.510	0.391	-0.260	0.795
Stensvold (2010)	-0.106	0.427	0.182	-0.942	0.731	-0.248	0.804
Levinger (2008)	-0.328	0.374	0.140	-1.062	0.405	-0.878	0.380
Yuan (2019)	-3.139	0.233	0.054	-3.595	-2.683	-13.495	0.000
	-0.907	0.625	0.390	-2.131	0.317	-1.452	0.146

Statistics for each study

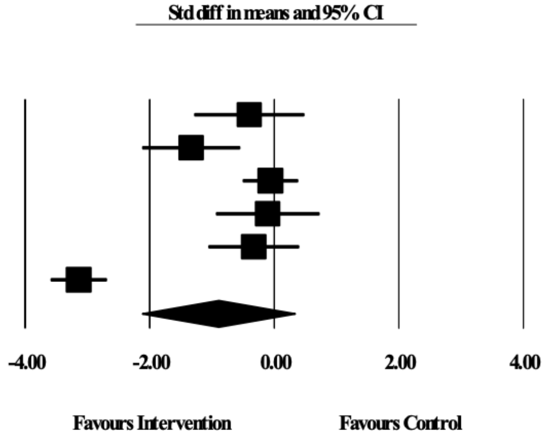

DBP Changes

Study name	Std diff in means	Standard error	Variance	Lower limit	Upper limit	Z-Value	p-Value
Alvarez (2012)	-0.159	0.450	0.203	-1.041	0.723	-0.353	0.724
DeVallance (2016)	-2.709	0.506	0.256	-3.701	-1.717	-5.351	0.000
Korkmaz (2018); Venojarvi (2013)	-0.271	0.231	0.053	-0.724	0.181	-1.176	0.240
Stensvold (2010)	-0.048	0.426	0.182	-0.884	0.788	-0.113	0.910
Levinger (2008)	-0.019	0.372	0.138	-0.747	0.710	-0.050	0.960
Yuan (2019)	-1.853	0.186	0.035	-2.218	-1.488	-9.954	0.000
	-0.834	0.445	0.198	-1.706	0.038	-1.875	0.061

Statistics for each study

Std diff in means and 95% CI

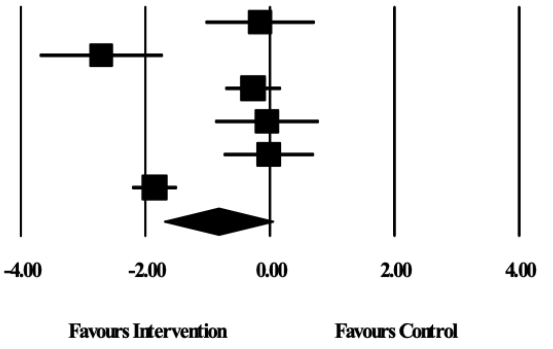

Total Cholesterol Changes

Study name	Std diff in means	Standard error	Variance	Lower limit	Upper limit	Z-Value	p-Value
Dai (2019)	-1.225	0.269	0.072	-1.752	-0.698	-4.559	0.000
Flandez (2017)	-0.302	0.318	0.101	-0.926	0.321	-0.951	0.342
Korkmaz (2018); Venojarvi (2013)	-3.000	0.335	0.112	-3.656	-2.344	-8.965	0.000
Stensvold (2010)	-0.183	0.427	0.183	-1.020	0.655	-0.427	0.669
Yuan (2019)	-0.727	0.161	0.026	-1.042	-0.412	-4.521	0.000
Yan (2019)	-0.171	0.239	0.057	-0.641	0.298	-0.716	0.474
	-0.933	0.371	0.138	-1.660	-0.206	-2.515	0.012

Statistics for each study

Std diff in means and 95% CI

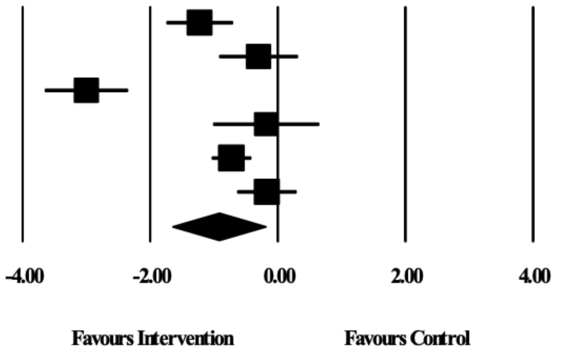

Triglyceride Changes

Study name	Std diff in means	Lower limit	Upper limit	p-Value
Dai (2019)	-0.406	-0.894	0.083	0.103
DeVallance (2016)	-1.500	-2.312	-0.688	0.000
Huffman (2014)	-0.371	-0.996	0.254	0.244
Korkmaz (2018); Venojarvi (2013)	-2.000	-2.551	-1.449	0.000
Stensvold (2010)	-0.181	-1.019	0.656	0.672
Levinger (2008)	-0.233	-0.964	0.498	0.532
Yuan (2019)	-0.628	-0.940	-0.315	0.000
Yan (2019)	-0.333	-0.805	0.139	0.167
	-0.705	-1.132	-0.279	0.001

Std diff in means and 95% CI

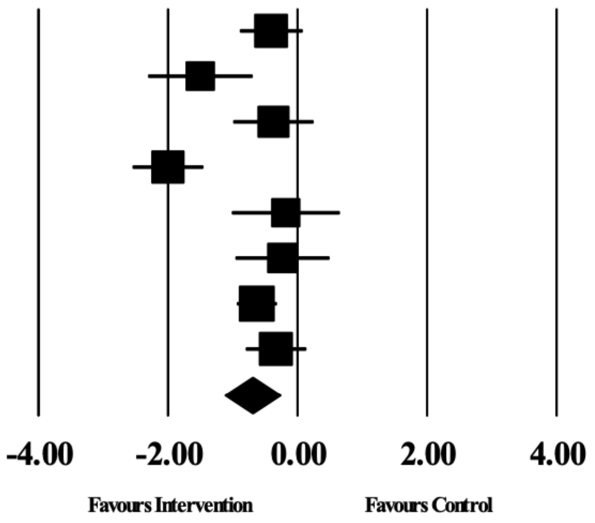

Waist Circumference Changes

Study name	Std diff in means	Standard error	Variance	Lower limit	Upper limit	Z-Value	p-Value
	Statistics for each study						
Alvarez (2012)	-0.166	0.450	0.203	-1.048	0.716	-0.369	0.712
Korkmaz (2018); Venojarvi (2013)	-1.281	0.252	0.064	-1.775	-0.787	-5.081	0.000
Levinger (2007)	-0.142	0.366	0.134	-0.858	0.575	-0.388	0.698
Mager (2008)	-0.476	0.361	0.131	-1.184	0.232	-1.318	0.188
Stensvold (2010)	-0.030	0.426	0.182	-0.866	0.806	-0.070	0.944
Levinger (2008)	-0.121	0.372	0.138	-0.850	0.608	-0.325	0.745
	-0.422	0.241	0.058	-0.894	0.050	-1.750	0.080

Std diff in means and 95% CI

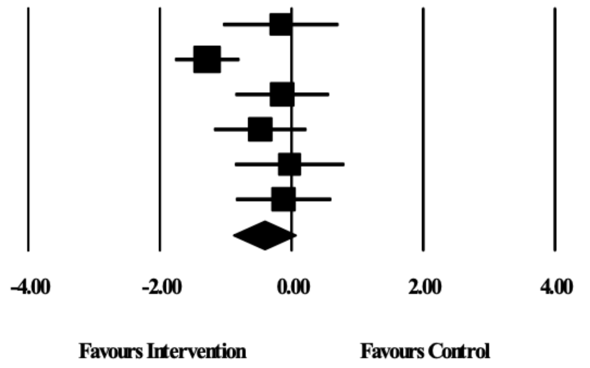

Declarations

Ethics approval and consent to participate Not applicable

Consent for publication Not applicable

Funding This study was supported through the Oakland University School of Health Sciences grant.

Competing interests Raza Qadir, Nicholas F. Sculthorpe, Taylor Todd, and Elise C. Brown declare that they have no competing interests.

Availability of data and material Data supporting the findings of this study are available from the corresponding author on request.

Author Contributions ECB and RQ designed the research and conducted the searches and screening. RQ and TT extracted the data, which were verified by ECB. NFS performed the statistical analyses. RQ wrote the manuscript with critical input from ECB and NFS.

Acknowledgements Not applicable.
